# Supplementary material for: Embryonic and aglomerular kidney development in the bay pipefish, Syngnathus leptorhynchus
Source: PLoS One. 2022 May 12;17(5):e0267932. doi: 10.1371/journal.pone.0267932 (PMC9098012; doi:10.1371/journal.pone.0267932)
Supplement: S1 Table — (DOCX) [file pone.0267932.s001.docx]

S1 Table.

| Embryonic stage | proteinase K (ug/ml) | permeabilization time (minutes) | permeabilization temperature (degrees celcius) | additional manipulations |
| --- | --- | --- | --- | --- |
| blastula/disc stages | 15 | 15 | 25 | removed from brood pouch |
| segmentation stages | 15 | 15 | 25 | removed from brood pouch |
| early pharyngula | 15 | 15 | 25 | removed from brood pouch |
| mid pharyngula | 15 | 15 | 25 | portion of tail removed |
| late pharyngula | 30 | 15 | 25 | anterior of head and half tail removed |
| early hatching stages | 125 | 15 | 60 | anterior of head and half tail removed |
| late hatching stages | 250 | 15 | 60 | anterior of head and half tail removed |

Stage specific modifications to the stand *in situ* hybridization protocol
